# Neurodevelopmental outcomes in children conceived by assisted reproductive treatment

**DOI:** 10.1192/j.eurpsy.2024.1596

**Published:** 2024-08-27

**Authors:** B. Abdelmoula, I. Bouaziz, A. Lamine, N. Bouayed Abdelmoula

**Affiliations:** Genomics of Signalopathies at the service of Precision Medicine - LR23ES07, Medical University of Sfax, Sfax, Tunisia

## Abstract

**Introduction:**

Impact of assisted reproductive treatment (ART) techniques on the child’s mental development is the focus of numerous studies. Whereas several studies have found potential negative effects on ART children’s psychosocial health, others recognized that the data on the cognitive and psychosocial development of ART children are comforting.

**Objectives:**

Here, we aim to state the current findings concerning psychological outcomes in children conceived by ART.

**Methods:**

Using as key words “assisted reproductive” and as filter “meta-analysis”, we comprehensively reviewed the scientific literature through new meta-analysis during the five last-years resuming the main conclusions of these studies to define principal through psychological conditions in children conceived by diverse ART techniques and approaches.

**Results:**

Our review showed that since 1978, the date of the first birth using in vitro fertilization technology (IVF), more than 10 million children are conceived by ART. Our research revealed 441 meta-analysis. After a comprehensive analysis of abstracts, only four meta-analysis were selected. Chronologically from 2019 to 2023, the first studies showed that the risk of intellectual disability and autism spectrum diseases (ASD) were higher in intra-cytoplasmic sperm injection (ICSI) children compared to conventional IVF children. The differences in the risk of neurodevelopmental disorders in children born after frozen and fresh embryo transfers were not significant. Analysis of potential cofounder effects such as multiple and preterm birth having a significant correlation with neurodevelopmental disorders suggested that ART is unlikely to cause negative impacts on children’s neurodevelopment. The findings of the most recent meta-analysis showed that the use of ART did not associate with the risk of ASD,

**Conclusions:**

Given the multitude of factors modulating ART, from the indication and parental background to the type of used technique and approach, the results of the studies that investigated the association between ART and neurodevelopmental outcomes remain yet contradictory.

**Disclosure of Interest:**

None Declared

